# The natural hybridization between species *Ligularia nelumbifolia* and *Cremanthodium stenoglossum* (Senecioneae, Asteraceae) suggests underdeveloped reproductive isolation and ambiguous intergeneric boundary

**DOI:** 10.1093/aobpla/plab012

**Published:** 2021-03-04

**Authors:** Li Hu, Rui Yang, Yue-Hua Wang, Xun Gong

**Affiliations:** 1 Plant Science Institute, School of Life Sciences, Yunnan University, Kunming 650201, Yunnan, China; 2 CAS Key Laboratory for Plant Diversity and Biogeography of East Asia, Kunming Institute of Botany, Chinese Academy of Sciences, Kunming 650201, China; 3 Key Laboratory of Economic Plants and Biotechnology, Kunming Institute of Botany, Chinese Academy of Sciences, Kunming 650201, China; 4 University of Chinese Academy of Sciences, Beijing 100049, China

**Keywords:** *Cremanthodium*, ddRAD-seq, F_1_s, *Ligularia*, natural hybridization

## Abstract

Natural hybridization is frequent in plants and is considered an important factor facilitating speciation. The natural intergeneric hybridization between *Ligularia* and *Cremanthodium* was previously confirmed using a couple of DNA markers. However, the mechanism of this intergeneric hybridization and the role of reproductive isolation in the process of hybridization remain unclear. Here we used double-digest restriction site-associated DNA sequencing (ddRAD-seq) to further quantify the occurrence of hybridization, the genetic structure of the hybrid population and the role of reproductive isolation between *Ligularia nelumbifolia* and *Cremanthodium stenoglossum*. The results based on the ddRAD-seq SNP data sets indicated that hybridization between *L. nelumbifolia* and *C. stenoglossum* was restricted to F_1_s, and no gene introgression was identified between these two species. STRUCTURE analysis and maximum likelihood (ML) tree results showed a slightly larger genetic contribution of *L. nelumbifolia* to putative hybrid F_1_s. We deduced that the reproductive isolation between these two parent species is not well-developed but still strong enough to maintain the genetic integrity of the species, and that their F_1_s are sterile or with low fertility. Given the poorly resolved phylogenetic relationship between *Ligularia* and *Cremanthodium*, the occurrence of natural hybridization between *L. nelumbifolia* and *C. stenoglossum* may provide new insights into the re-circumscription and re-delimitation of these two genera.

## Teaser

Natural hybridization is common in plants and results in different evolutionary consequences for hybridizing species. Here, we used ddRAD-seq to explore the natural hybridization between *Ligularia nelumbifolia* and *Cremanthodium stenoglossum*. The hybridization between these two species is an early hybridization event, and their hybrids were limited to the F_1_ generation and therefore species genetic integrity might be maintained. Compared to the parent species *C. stenoglossum*, *L. nelumbifolia* made a slightly larger genetic contribution to their putative hybrid F_1_ generation. The hybridization between these two species provides a new insight into the re-circumscription and re-delimitation of these two genera.

## Introduction

Natural hybridization occurs in ~25 % of plant species (Folk *et al.* 2018), and is considered an important factor facilitating speciation and innovation through the transfer of adaptive traits via introgression, the formation of recombinant forms, and allopolyploid or homoploid hybridization (Mallet 2007; Abbott *et al.* 2010, 2013). Homoploid hybridization between species continually produces hybrids of mixed ancestry in hybrid zones (Harrison 1993; Payseur 2010). The geographic areas where hybridization is localized are called hybrid zones (Barton and Hewitt 1985). Hybrid individuals consist of early- or later-generation hybrids in hybrid zones. In some cases, hybrid swarms (various recombinant types) are found in hybrid zones (Barton and Hewitt 1985; Abbott 2017).

The Qinghai–Tibetan Plateau (QTP) is the highest and one of the most extensive plateaus in the world, and has been considered as one of the most important biodiversity hotspots in the world (Myers *et al.* 2000). Natural hybridization has been suggested to have contributed to the high species diversity on the QTP and adjacent areas because of frequent secondary sympatry during relatively stable stages between different uplifts of the QTP (Wang *et al.* 2001; Liu *et al.* 2006). As a consequence, hybridization within hybrid zones was proposed to have played an important role in several diversified plant groups in the QTP region, such as Rhododendron (Ma *et al.* 2010; Marczewski *et al.* 2015), Primula (Ma *et al.* 2014), Roscoa (Du *et al.* 2012) and Silene (Zhang *et al.* 2016b).

*Ligularia* and *Cremanthodium*, belonging to the subtribe Tussilagininae (Senecioneae, Asteraceae), are both perennial alpine plants and share several morphological traits (Liu 1989; Chen *et al.* 2011). The primary morphological difference between *Ligularia* and *Cremanthodium* involves the shape of the involucre, which is cylindrical for *Ligularia* and campanulate for *Cremanthodium* (Liu and Illarionova 1989). Both genera are non-monophyletic, clustering into the *Ligularia–Cremanthodium–Parasenecio* (L–C–P) complex, a fairly diversified clade that contains more than 200 species that are endemic to the QTP in eastern Asia (Liu *et al.* 2006; Ren 2012). Traditionally, *Cremanthodium* species were regarded as alpine derivatives of *Ligularia* species (Wulff 1944; Drury 1966); however, they are still recognized as two genera. Given the close relationship and high diversity of *Ligularia* and *Cremanthodium* on the QTP (Liu *et al.* 2006), it can be speculated that hybridization might also play a role in their respective diversification and ambiguous phylogeny. However, the hybridization reported within these two genera is asymmetric. In *Ligularia*, many species occur together with overlapping flowering periods, and natural hybridization has frequently been observed (Pan *et al.* 2008; Yu *et al.* 2011, 2014a, b; Pante *et al.* 2015; Zhang *et al.* 2018a), whereas little is known about hybridization in *Cremanthodium*. In recent years, studies on *Cremanthodium* have focused on its chemical composition and the description of new species (Wang *et al.* 2018; Fei *et al.* 2019; Tori *et al.* 2019). Meanwhile, intergeneric hybridization within Asteraceae is rarely reported. Although *Ligularia* is closely related to *Cremanthodium* in morphology and phylogeny, little information is available on natural hybridization between *Ligularia* and *Cremanthodium*.

The natural hybridization between *Ligularia* and *Cremanthodium* was recently confirmed by a couple of DNA markers in a pilot study (Ning *et al.* 2019). The results showed that a high frequency of the F_1_ class and relatively fewer backcrossing individuals might prevent the formation of new species with a bidirectional hybrid origin. However, both the genetic structure of the hybrid progeny population and the role of reproductive isolation in the process of hybridization remain unclear. Here we extended the previous study by using double-digest restriction site-associated DNA (ddRAD) sequencing to quantify the extent of hybridization in this hybrid zone. Double-digest restriction site-associated DNA can generate massive genome-wide single-nucleotide polymorphism (SNP) data sets, without any prior genomic information, thus making it widely applied to evolutionary studies involving resolving long-standing taxonomic problems, species boundaries in taxonomically complex groups, population genetics and phylogenetic research (Wagner *et al.* 2017, 2020; Spriggs *et al.* 2019; Khan *et al.* 2020). Two taxa were included in this study: *Cremanthodium stenoglossum* grows mainly at an altitude of 3700–5000 m in the southern part of Qinghai province and northwest Sichuan province, while *Ligularia nelumbifolia* mainly occurs at an altitude of 2400–3900 m in Northwest to Northeast Yunnan, Southwest to Northwest Sichuan, West Hubei and Southwest Gansu (Chen *et al.* 2011). The investigated hybrid zone is located in Northwest Sichuan **[see**  **Supporting Information—Fig. S1****]**, where the altitude is about 4600 m.

In this study, we aimed to combine the genome-wide SNP data obtained from ddRAD sequencing and field investigation to address two questions: (i) how and to what extent does intergeneric hybridization occur; and (ii) can the genetic data offer information to help understand the genetic structure of the hybrid populations and the mechanism of reproductive isolation.

## Methods

### Plant sampling and DNA extraction

In this study, two sympatric species *L. nelumbifolia* and *C. stenoglossum* were selected as the study taxa from Ganzi County, Sichuan province of China, where the average altitude is about 4600 m **[see**  **Supporting Information—Fig. S1]**. To date, this hybrid zone is the only one found in our field investigation. Young leaves of 80 accessions from *L. nelumbifolia*, *C. stenoglossum* and their hybrid populations were randomly collected, with 20 individuals for each taxon. The materials were dried immediately in silica gel and genomic DNA was extracted using the modified CTAB protocol (Doyle 1991). The primary morphological difference between hybrid populations or morphologically intermediate individuals produced by *L. nelumbifolia* and *C. stenoglossum*, here designated as morphotypes G and P, is the colour of the leaf margin and leaf stalks, where G is green, and P is purple **[see**  **Supporting Information—Fig. S2****]**.

### Library preparation, sequencing and processing of RAD-seq data

Library preparation followed the protocol of Peterson *et al.* (2012). Genomic DNA of each individual was digested with two restriction enzymes: MspI (cut site 5′-CCGG-3′) and AvaII (cut site 5′-GWCC-3′); fragments were size-selected to a range of 500–700 bp. Libraries were pooled to a target of 5 Gb raw data per individual (Zhang *et al.* 2018a). Double-digest restriction site-associated DNA sequencing was carried out by Jiakang Bio Technologies Corporation (Wuhan, China), and was performed on an Illumina HiSeq 4000 System (San Diego, CA, USA) with 150-bp paired-end reads.

The *de novo* pipeline in STACKS v.2.52 (Catchen *et al.* 2011, 2013; Rochette *et al.* 2019) was implemented to identify and obtain SNP data sets. Raw reads were de-multiplexed and quality-filtered using *process_radtags*. During the process, all reads were trimmed to 140 bp, and low-quality bases at the end of sequences and reads with ambiguous or low-quality bases (below a Phred score of Q10) were discarded. Read quality and Guanine cytosine (GC) content were assessed by FastQC v.0.11.8 (Andrews 2017). Since the number of clean reads of Ln_19 and G_19 was extremely low **[see**  **Supporting Information—Table S2****]**, we then removed these two samples from the analyses in order to maintain the integrity of the data set. As a result, 78 samples were used in the subsequent analysis.

For Stacks analysis, we implemented *denovo_map.pl* to analyse each sample, which executes the Stacks pipeline by running *ustacks*, *cstacks* and *sstacks*. The deleveraging algorithm (d) and removal algorithm (r) were implemented to remove highly repetitive stacks and over-merged tags. The minimum depth of coverage required to create a stack (m) was set to five, and the maximum nucleotide mismatch allowed between stacks (M) was two. In *cstacks*, which creates a catalogue of all loci across the population (or from just the parents if processing a genetic map), we set the catalogue of loci built with the mismatch number allowed between sample tags (n) as four. Finally, the SNP data set was generated in the populations pipeline with the genotype of each individual for every polymorphic site. A locus had to be present in at least 75 % of the individuals (*r* = 0.75) and in each of the four populations (*p* = 4) as the optimal parameters.

### Hybridization detection based on SNP data sets

Genetic admixture of all individual samples was estimated using a Bayesian assignment analysis implemented in STRUCTURE v.2.3.4 (Pritchard *et al.* 2000). *K* was set to range from 1 to 10 with 10 replicates per *K*. The run was set for 25 000 steps of burn-in followed by 100 000 Markov chain Monte Carlo algorithms (MCMC) iterations. No population priors were applied in the analyses. The optimal *K* value was identified using STRUCTURE HARVESTER (Earl 2012). Admixture proportions of 10 repeats per *K* value were averaged using CLUMPP v.1.1.2 (Jakobsson and Rosenberg 2007) and the results were plotted by DISTRUCT v.1.1 (Rosenberg 2004).

A Bayesian clustering method was applied in NEWHYBRIDS v.1.1 (Anderson and Thompson 2002) to calculate the posterior probability of surveyed individuals assigned to different hybrid categories, i.e. Pure parental 1 (P1), Pure parental 2 (P2), F_1_ class (F_1_), F_2_ class (F_2_), backcross to P1 (BCP1) or backcross to P2 (BCP2). Given the limitation of the NEWHYBRIDS software, which can only analyse about 400 SNPs, the SNPs were randomly selected using a python script. The program ran for 100 000 MCMC iterations with 25 000 iterations as burn-in.

We used raxmlGUI v.1.3 (Silvestro and Michalak 2012), which is the graphical interface of RAxML v.8.2.12 (Stamatakis 2006; Stamatakis *et al.* 2008), to construct the maximum likelihood (ML) tree in order to assess the phylogenetic relationships of 78 samples. Three individuals of *L. subspicata* were employed as the outgroup, and the GTR + G nucleotide substitution model was applied in the ML tree inference with 1000 bootstrap replicates (rapid bootstrap algorithm). A principal coordinate analysis (PCoA) was conducted for all individuals using GenAlex v.6.5 (Peakall and Smouse 2012) and pairwise *F*_ST_ values for parental species and morphotypes of hybrids were calculated using Arlequin v.3.5 (Excoffier and Lischer 2010).

## Results

### ddRAD sequencing

After quality filtering, we generated 1.2 billion first (left) paired-end reads in total (we only applied the single-end reads for analyses) of 80 individuals. On average, 15.3 million reads were processed per individual for these 80 individuals, ranging from 0.17 million (G19) to 55.5 million (Cs20) **[see**  **Supporting Information—Table S1****]**. The average GC content was 44 %, without apparent differences among investigated individuals. After removing two individuals with extremely low reads data, the retained reads from 78 individuals were assembled into an average of 310 880 putative loci or RAD tags, per individual, with the average coverage depth per tag of 15.3× **[see**  **Supporting Information—Table S2****]**. A catalogue of 21 280 991 putative loci was constructed by *cstacks* and an average of 272 833 putative loci, per individual, was matched to the catalogue in *sstacks*. The final SNP data set included a total of 2540 sites.

### Genetic structure and genomic differentiation

**Figure 1.**
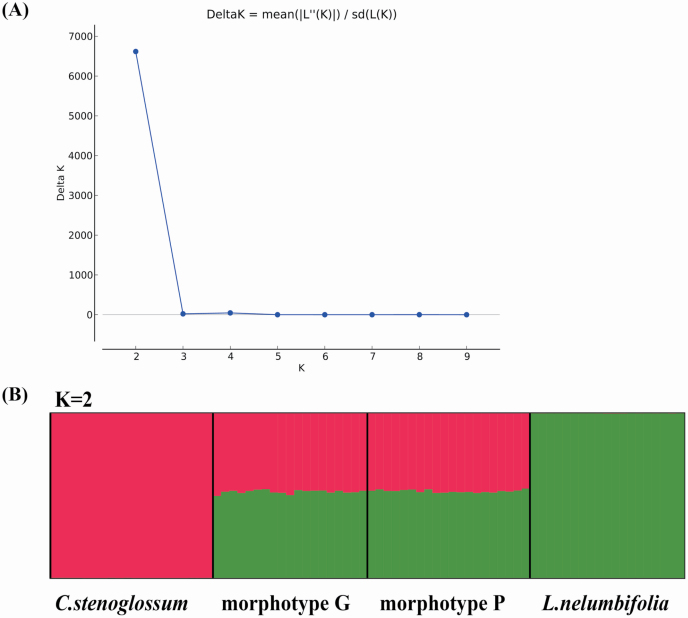
STRUCTURE plots for different data sets 2540 SNPs, 78 individuals. (A) Rate of change in the log probability of data between successive *K* values. (B) Genetic admixture of 78 individuals when *K* = 2.

The STRUCTURE analysis based on 2540 SNPs showed that when *K* = 2, the posterior log probability of the data, *L*(*K*), achieved the largest increase and the delta *K* value was the maximum (Fig. 1A), suggesting that all 78 investigated individuals could be divided into two genetic clusters (Fig. 1B), with *L. nelumbifolia* and *C. stenoglossum* constituting pure groups. In contrast, all hybrid individuals of morphotype G and P possessed mixed genetic components from the two groups. The *q* value of hybrids showed that about 52 % of the genetic component was from the parent *L. nelumbifolia* and about 48 % was from the parent *C. stenoglossum*.

The genetic differentiation coefficient *F*_ST_ value was highest between *L. nelumbifolia* and *C. stenoglossum* (*F*_ST_ = 0.73802; Table 1), and the *F*_ST_ values between hybrids and the parental species were consistent. We also mixed the morphotype G and P as one population to analyse the *F*_ST_ with their parent species **[see**  **Supporting Information—Table S3****]**, and obtained similar results.

**Table 1.** Pairwise *F*_ST_ values for *L. nelumbifolia*, *C. stenoglossum* and two hybrids morphotypes based on 2540 SNPs.

	*C. stenoglossum*	Morphotype G	Morphotype P	*L. nelumbifolia*
*C. stenoglossum*	*	0.00000 + −0.0000	0.00000 + −0.0000	0.00000 + −0.0000
Morphotype G	0.2627	*	0.11914 + −0.0129	0.00000 + −0.0000
Morphotype P	0.26425	−0.01587	*	0.00000 + −0.0000
*L. nelumbifolia*	0.73802	0.29181	0.29188	*

**Figure 2.**
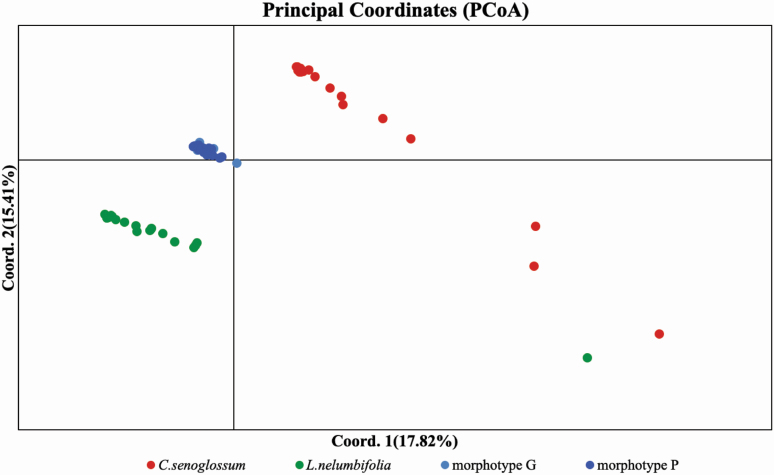
Principal coordinate analysis of all 78 individuals in this study. The first principal component explains 17.82 % of the variance and the second principal component explains 15.41 % of the variance.

Principal coordinate analysis identified three well-differentiated clusters (Fig. 2), corresponding to two parental species *L. nelumbifolia* and *C. stenoglossum*, and hybrids generated from *L. nelumbifolia* × *C. stenoglossum* (mixed morphotypes G and P). The distribution of several individuals from *L. nelumbifolia* and *C. stenoglossum* was scattered, which might be due to high genetic differences among individuals.

**Figure 3.**
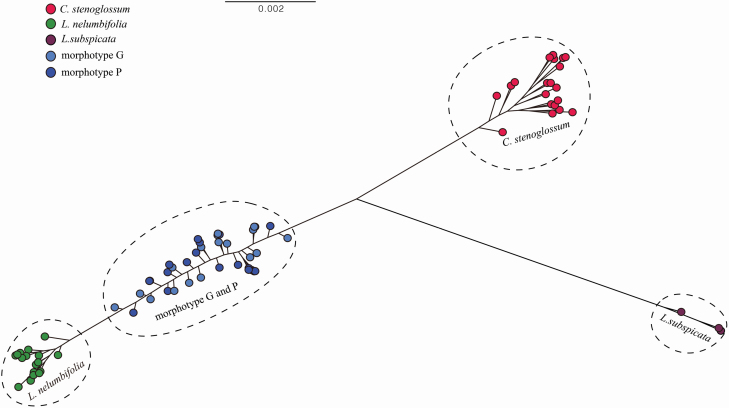
Maximum likelihood tree for 81 individuals (including 78 investigated individuals and three outgroup individuals) using RAxML.

The best tree from a full ML search showed that the 78 investigated individuals and three outgroup individuals formed four clades with high bootstrap values (Fig. 3). Individuals of two parental species (*L. nelumbifolia*, *C. stenoglossum*) as well as the outgroup *L. subspicata* were separately recovered as clades. Furthermore, the morphotypes G and P were closer to *L. nelumbifolia*, which was consistent with the results of STRUCTURE.

**Figure 4.**
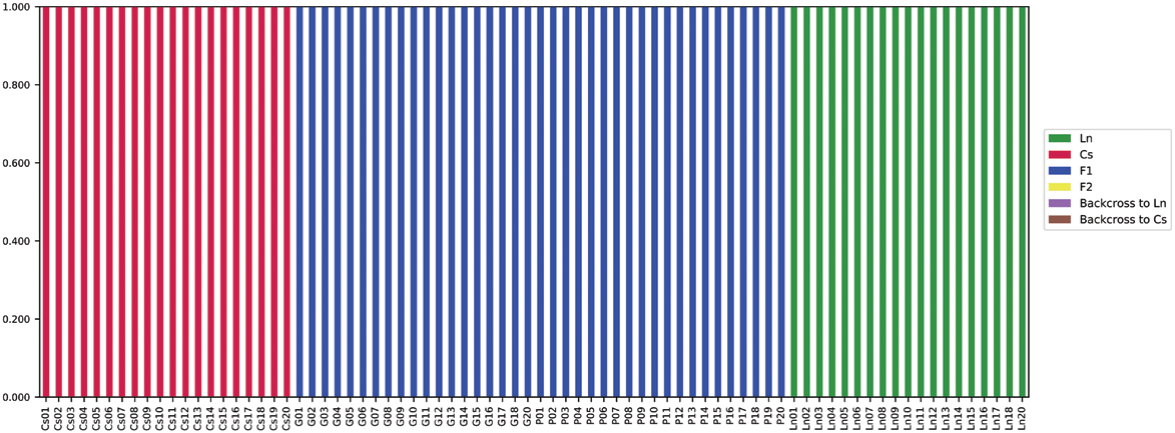
Genotype class assignment of 78 individuals in hybridization group *L. nelumbifolia* × *C. stenoglossum* by NEWHYBRIDS based on 400 SNPs. Ln = *L. nelumbifolia*; Cs = *C. stenoglossum*; G = morphotype G; P = morphotype P.

NEWHYBRIDS analysis indicated that individuals of *L. nelumbifolia* and *C. stenoglossum* were assigned to one parent, respectively (Fig. 4), consistent with morphological identification. Meanwhile, hybrid individuals were all robustly assigned to the F_1_ class (*P* = 1.000).

## Discussion

### The occurrence of natural hybridization

Hybridization is generally thought to be limited in genetically similar, closely related species (Abbott 2017). This has been demonstrated for some plant genera and animal species (Moyle *et al.* 2004; Scopece *et al.* 2008; Roux *et al.* 2016). Hybridization within hybrid zones has been proposed to have played an important role in the spectacular radiations of the L–C–P complex (Liu *et al.* 2006). Within the QTP region, at least six sets of natural hybridization within nine *Ligularia* species have been reported (Pan *et al.* 2008; Yu *et al.* 2011, 2014a, b; Ning *et al.* 2017; Zhang *et al.* 2018a, b). However, natural hybridization studies in *Cremanthodium* are scarce. The hybridization between *L. nelumbifolia* and *C. stenoglossum* has been confirmed by DNA data and their chemical composition (Ning *et al.* 2019; Okamoto *et al.* 2019). Unique chemical compounds (eremophilane sesquiterpenes) were found in both hybrids and were deduced to have come from their *Cremanthodium* parents, as sesquiterpenes were detected in *C. stenoglossum* but not in *L. nelumbifolia* (Okamoto *et al.* 2019). Natural hybridization between *C. stenoglossum* and *L. nelumbifolia* may be related to geographical sympatry, overlapping flowering periods, common pollinators and homoploidy. *Cremanthodium stenoglossum* blooms from July to August and *L. nelumbifolia* blooms from July to September (Liu 1989). Field observations showed that nectar-collecting insects had no species preference between *L. nelumbifolia* and *C. stenoglossum* and shuttled frequently among flowers in the study area. Karyotype analyses showed *L. nelumbifolia* and *C. stenoglossum* were both diploid with 2n = 58 (Liu *et al.* 2001; Ren 2012), which makes homoploid hybridization possible (Kou *et al.* 2017). Combining the ambiguous morphological differences and phylogeny between *Ligularia* and *Cremanthodium*, the above ecological factors further provided possibilities for natural hybridization between *L. nelumbifolia* and *C. stenoglossum*, supporting the view that *Cremanthodium* species are alpine derivatives of *Ligularia* species (Wulff 1944; Drury 1966). Although the morphology and phylogeny of *Ligularia* and *Cremanthodium* are unclear, the current study may offer a new insight into their relationship by sampling the potential hybrids during the field survey.

### Genetic structure of the F_1_s

Our previous study based on four nuclear DNA and three chloroplastic DNA fragments as molecular evidence proved the natural hybridization between *L. nelumbifolia* and *C. stenoglossum*, and only F_1_s were detected (Ning *et al.* 2019). In this study, we further quantified the genetic composition of the hybrids, with the results of the STRUCTURE analysis showing that all hybrids have mixed genetic components, of which 52 % are from *L. nelumbifolia* and 48 % from *C. stenoglossum* (Fig. 1B); the ML tree showed that the hybrids clustered together and were closer to the parent *L. nelumbifolia* (Fig. 3); the results of NEWHYBRIDS showed that all hybrids were detected as F_1_s and that the parents were a pure group (Fig. 4). All these results supported our previous study demonstrating natural hybridization between *L. nelumbifolia* and *C. stenoglossum*, and the detection of only F_1_s in this hybrid zone. Additionally, no significant genetic differences were detected between morphotypes G and P, suggesting that morphological characters cannot be used to distinguish the F_1_s or are not imprinted in the ddRAD-based SNPs. Although there were very small differences in genetic components between the hybrids and their parent species, we considered that *L. nelumbifolia* might contribute slightly more to the natural hybridization.

### Assumptions about reproductive isolation

It is reasonable to infer that the natural hybridization that has occurred between *L. nelumbifolia* and *C. stenoglossum* may be an early event in natural hybridization, since hybrids normally need a long time to establish as a new species and produce isolation barriers with their parental species. In the hybrid zone, some of the hybrid offspring were still flowering, and we investigated most of the individuals that seemed to have seeds, but no seed was found in their infructescence. We made two assumptions based on this situation. The first was that the F_1_s were sterile. In our previous studies on the natural hybridization of *Ligularia* species, the seeds collected from F_1_s were usually few in number, and most were sterile (Pan *et al.* 2008; Yu *et al.* 2011; Zhang *et al.* 2018a). In the current case, F_1_ individuals can develop into complete adults, and there must be strong reproductive isolation between F_1_s and their parents to hinder seed formation. Since post-zygotic mechanisms include hybrid inviability, hybrid sterility and the failure or reduction of successful reproduction in subsequent generations, we propose that post-zygotic isolation between *L. nelumbifolia* and *C. stenoglossum* is the mechanism causing reproductive isolation between these two species (Milne and Abbott 2008; Pellegrino *et al.* 2009; Ishizaki *et al.* 2013).

The second assumption was that the F_1_s were fertile. However, as no seed could be found in the wild, this would imply low fertility. Since no backcrossing individual was detected in this study (Fig. 4), there is a high possibility that there were strong isolation barriers between their parent species.

Based on the above assumptions, we propose that reproductive isolation is not well-developed but is still strong between *L. nelumbifolia* and *C. stenoglossum*. This may be one of the reasons for the lack of later-generation hybrids, and it is common compared to other hybridization events reported in *Ligularia*. For instance, *Ligularia* × *maoniushanensis*, a natural hybrid of *L. paradoxa* and *L. duciformis*, showed abnormal meiotic behaviour and inviability of seeds. Inter-simple sequence repeat (ISSR) markers indicated that all observed hybrids were the F_1_ generation, implying the sterility of F_1_s (Pan *et al.* 2008). In addition, hybrids of *L. nelumbifolia* and L. subspicata were mostly composed of F_1_s and their seed germination rate is extremely low (Yu *et al.* 2011). Similarly, the hybridization between *L. cyathiceps* and *L. duciformis*, *L. yunnansis* and *L. duciformis* showed the same results with most hybrids identified as F_1_s (Zhang *et al.* 2018a). The observation of few F_1_ hybrids may be evidence for the existence of strong reproductive isolation, as the theoretical expectation of F_1_s individuals is 25 % if there are equal numbers of parental individuals practicing a selfing rate of 0.5 but outcrossing at random with respect to taxon, and assuming no reduction in F_1_ viability (Twyford *et al.* 2015). This was the case between *L. nelumbifolia* and *C. stenoglossum*, where hybrids of morphotype G and P were rarer compared to abundant parental individuals in the field.

### Future directions

The only hybrid zone of *L. nelumbifolia* and *C. stenoglossum* has been discovered several years. However, a lot of questions remain unanswered. The genetic data alone are not sufficient to illustrate the reproductive barriers between these two species. Studies focusing on pre-pollination including niche differentiation, phenological isolation and pollinator specialization, as well as post-pollination including pollen–stigma interactions and pollen competition, hybrid seed formation, F_1_ fertility and viability, and F_2_ fertility and viability are needed.

At present, *L. nelumbifolia*, *C. stenoglossum* and their hybrids are growing together, with the parent species in a dominant region of the hybrid zone. Although we divided the hybrid population into two morphotypes G and P, the genetic results showed that there was no difference between them, and the mechanism leading to this situation remains unknown. According to the Flora of China (Chen *et al.* 2011), this hybrid zone (latitude 4600 m) is much higher than the distribution of *L. nelumbifolia* (below 4000 m). It is possible that factors related to climate change, human interference or something else have promoted the migration of *L. nelumbifolia*, which has shifted its distribution to a higher altitude. In this situation it may have accidentally interbred with the alpine *C. stenoglossum* and the hybrids have remained at the early phase. Infrageneric hybridization between taxa from different altitudes has been reported, such as the hybridization in an altitudinal hybrid zone on Mount Etna between two ragwort species (the low elevation *Senecio chrysanthemifolius* and high elevation *S. aethnensis*) (Brennan *et al.* 2009), whereas our reported intergeneric hybridization event is possibly rather sporadic, since no other similar case has been reported within the Asteraceae from the QTP.

Since natural hybridization may drive rare taxa to extinction through genetic and demographic swamping (Rhymer and Simberloff 1996; Huxel 1999; Todesco *et al.* 2016), our results could raise attention about the future of this area; conversely, a net fitness could be gained by one or both taxa without loss of species integrity by adaptive trait transfer between species by introgression (Abbott *et al.* 2016; Lamichhaney *et al.* 2016; Zhang *et al.* 2016a). Future studies should focus on the dynamic changes of taxa composition in this hybrid zone, and field work will be essential for seed collection and the discovery of hybrid zones involving the two species from the other regions.

## Conclusions

Based on the evidence from genetic data and field investigation, this study demonstrates the natural hybridization between *L. nelumbifolia* and *C. stenoglossum* in the eastern QTP and adjacent areas, with the hybrid population found to be composed of the F_1_ generation. The occurrence of hybridization between these species may be related to some ecological and physiological factors such as geographical sympatry, sufficient population size, overlapping flowering periods, common pollinators and homoploidy. Whole genomic-associated genetic data suggested that *L. nelumbifolia* may contribute slightly more to the putative hybrid F_1_s than the other parent. To date, this is the only hybrid zone found in the wild, F_1_s were sterile or with low fertility and little backcrossing was detected, implying that reproductive barriers between these two species are not well-developed but still strong enough to maintain the genetic integrity of the species. Since natural hybridization may be a common phenomenon in the wild, this study will help us to further understand the formation and the maintenance of hybrid zones. Moreover, although the morphology and phylogeny between *Ligularia* and *Cremanthodium* remain unclear, this study offers new insights into their intergeneric relationship.

## Supporting Information

The following additional information is available in the online version of this article—

**Figure S1.** Geographic location of the hybrid zone and the distribution of L. nelumbifolia and C. stenoglossum. Green circle is the distribution area of L. nelumbifolia; red circle is the distribution area of C. stenoglossum; black point is the location of hybrid zone.

**Figure S2.** Habitats, leaf and flower characteristics of *L. nelumbifolia* (a–c), putative hybrids morphotype P (d–f), morphotype G (g–i) and *C. stenoglossum* (j–l) investigated in this study.

**Table S1.** Statistics describing cleaned reads for each investigated individual after quality filtering using *process_radtags*.

**Table S2.** Summary of loci recovered for each investigated sample in *ustacks* and number of matched loci in *sstacks*.

**Table S3.** Pairwise *F*_ST_ values for *L. nelumbifolia*, *C. stenoglossum* and hybrid population (combines morphotypes G & P) based on 2540 SNPs.

plab012_suppl_Supplementary_Materials

## Notes

**Evolution & Diversity. Chief Editor:** Jeremy Beaulieu
