# Supplementary material for: The natural hybridization between species Ligularia nelumbifolia and Cremanthodium stenoglossum (Senecioneae, Asteraceae) suggests underdeveloped reproductive isolation and ambiguous intergeneric boundary
Source: AoB Plants. 2021 Mar 4;13(2):plab012. doi: 10.1093/aobpla/plab012 (PMC7994929; doi:10.1093/aobpla/plab012)
Supplement: plab012_suppl_Supplementary_Materials [file plab012_suppl_supplementary_materials.docx]

**Supporting Information**

**Figure S1.** Geographic location of the hybrid zone and the distribution of L. nelumbifolia and C. stenoglossum. Green circle is the distribution area of L. nelumbifolia; Red circle is the distribution area of C. stenoglossum; black point is the location of hybrid zone.

**Figure S2**. ﻿Habitats, leaf and flower characteristics of L. nelumbifolia (a-c), putative hybrids morphotype P (d-f), morphotype G (g-i) and C. stenoglossum (j-l) investigated in this study.

**Table S1.** Statistics describing cleaned reads for each investigated individual after quality filtering using *process_radtags*.

**Table S2.** Summary of loci recovered for each investigated sample in ustacks and number of matched loci in sstacks.

**Table S3.** Pairwise F_ST_ values for L. nelumbifolia, C. stenoglossum and hybrids population (combines morphotypes G&P) based on 2,540 SNPs.

**Table S1.** Statistics describing cleaned reads for each investigated individual after quality filtering using *process_radtags*.

| Sample ID | Number of clean reads | Sequence length (bp) | GC% |
| --- | --- | --- | --- |
| Cs01 | 2414306 | 140 | 43 |
| Cs02 | 6053012 | 140 | 44 |
| Cs03 | 5694208 | 140 | 45 |
| Cs04 | 13861342 | 140 | 44 |
| Cs05 | 6378578 | 140 | 44 |
| Cs06 | 7877930 | 140 | 44 |
| Cs07 | 35787920 | 140 | 44 |
| Cs08 | 1086065 | 140 | 43 |
| Cs09 | 48165592 | 140 | 44 |
| Cs10 | 29980152 | 140 | 49 |
| Cs11 | 16479488 | 140 | 44 |
| Cs12 | 15502065 | 140 | 43 |
| Cs13 | 16165956 | 140 | 44 |
| Cs14 | 10567577 | 140 | 45 |
| Cs15 | 9439930 | 140 | 44 |
| Cs16 | 14644600 | 140 | 46 |
| Cs17 | 19989655 | 140 | 44 |
| Cs18 | 12200758 | 140 | 45 |
| Cs19 | 26041302 | 140 | 44 |
| Cs20 | 55157362 | 140 | 43 |
| Ln01 | 6434209 | 140 | 44 |
| Ln02 | 7411599 | 140 | 44 |
| Ln03 | 5678620 | 140 | 46 |
| Ln04 | 8593972 | 140 | 44 |
| Ln05 | 8132533 | 140 | 45 |
| Ln06 | 7019982 | 140 | 48 |
| Ln07 | 8392483 | 140 | 44 |
| Ln08 | 8884157 | 140 | 45 |
| Ln09 | 6448445 | 140 | 44 |
| Ln10 | 9523210 | 140 | 45 |
| Ln11 | 5156386 | 140 | 44 |
| Ln12 | 1700136 | 140 | 48 |
| Ln13 | 8560328 | 140 | 46 |
| Ln14 | 6514658 | 140 | 45 |
| Ln15 | 8686459 | 140 | 45 |
| Ln16 | 4201844 | 140 | 45 |
| Ln17 | 4537915 | 140 | 44 |
| Ln18 | 6404697 | 140 | 49 |
| Ln19 | 289672 | 140 | 56 |
| Ln20 | 8214213 | 140 | 45 |
| G01 | 11894337 | 140 | 44 |
| G02 | 17245916 | 140 | 44 |
| G03 | 21003790 | 140 | 43 |
| G04 | 14948762 | 140 | 43 |
| G05 | 37772363 | 140 | 44 |
| G06 | 16940235 | 140 | 43 |
| G07 | 17031989 | 140 | 44 |
| G08 | 46286656 | 140 | 43 |
| G09 | 29466307 | 140 | 45 |
| G10 | 23778654 | 140 | 45 |
| G11 | 18817875 | 140 | 44 |
| G12 | 17216842 | 140 | 44 |
| G13 | 21215540 | 140 | 43 |
| G14 | 26897621 | 140 | 43 |
| G15 | 17628931 | 140 | 45 |
| G16 | 21792863 | 140 | 43 |
| G17 | 17128209 | 140 | 43 |
| G18 | 9536986 | 140 | 45 |
| G19 | 179136 | 140 | 48 |
| G20 | 18328685 | 140 | 45 |
| P01 | 11718365 | 140 | 43 |
| P02 | 17746792 | 140 | 45 |
| P03 | 12548118 | 140 | 44 |
| P04 | 12386790 | 140 | 43 |
| P05 | 15722872 | 140 | 44 |
| P06 | 15575116 | 140 | 43 |
| P07 | 36451803 | 140 | 43 |
| P08 | 12219291 | 140 | 44 |
| P09 | 11582400 | 140 | 44 |
| P10 | 21352273 | 140 | 44 |
| P11 | 13916659 | 140 | 43 |
| P12 | 9481323 | 140 | 43 |
| P13 | 8349023 | 140 | 44 |
| P14 | 51364666 | 140 | 42 |
| P15 | 13391529 | 140 | 44 |
| P16 | 11953613 | 140 | 43 |
| P17 | 14805512 | 140 | 43 |
| P18 | 14097301 | 140 | 43 |
| P19 | 17679005 | 140 | 43 |
| P20 | 15138226 | 140 | 44 |

**Table S2.** Summary of loci recovered for each investigated sample in ustacks and number of matched loci in sstacks.

| Sample ID | Number of stacks | Mean coverage depth | | Std Dev | | Number of matched stacks | |
| --- | --- | --- | --- | --- | --- | --- | --- |
| Cs_01 | 33752 | | 15.93x | | 36.14 | | 31993 |
| Cs_02 | 99826 | | 15.51x | | 36.15 | | 92619 |
| Cs_03 | 95519 | | 14.83x | | 30.42 | | 87775 |
| Cs_04 | 235978 | | 14.94x | | 24.9 | | 212090 |
| Cs_05 | 119826 | | 13.78x | | 25.81 | | 109782 |
| Cs_06 | 158833 | | 14.59x | | 25.47 | | 144967 |
| Cs_07 | 476170 | | 21.51x | | 34.85 | | 428676 |
| Cs_08 | 10893 | | 18.41x | | 44.97 | | 10541 |
| Cs_09 | 714698 | | 24.19x | | 35.44 | | 633275 |
| Cs_10 | 509825 | | 21.75x | | 34.45 | | 460737 |
| Cs_11 | 337432 | | 16.12x | | 24.81 | | 303487 |
| Cs_12 | 353809 | | 15.46x | | 22.27 | | 316691 |
| Cs_13 | 317233 | | 15.23x | | 25.89 | | 283739 |
| Cs_14 | 191876 | | 15.39x | | 30.44 | | 177728 |
| Cs_15 | 171101 | | 14.47x | | 27.45 | | 156416 |
| Cs_16 | 267577 | | 16.30x | | 27.97 | | 241947 |
| Cs_17 | 348915 | | 16.11x | | 28.55 | | 311662 |
| Cs_18 | 230035 | | 15.24x | | 26.99 | | 207275 |
| Cs_19 | 441573 | | 19.29x | | 29.17 | | 395076 |
| Cs_20 | 782661 | | 25.72x | | 36.75 | | 693906 |
| Ln_01 | 100662 | | 15.34x | | 40.09 | | 92469 |
| Ln_02 | 109987 | | 15.29x | | 37.65 | | 98923 |
| Ln_03 | 98813 | | 13.86x | | 26.81 | | 88399 |
| Ln_04 | 155421 | | 14.04x | | 31.84 | | 138996 |
| Ln_05 | 138701 | | 14.40x | | 35.17 | | 125174 |
| Ln_06 | 90001 | | 14.73x | | 41.88 | | 81792 |
| Ln_07 | 142942 | | 14.64x | | 37.58 | | 128760 |
| Ln_08 | 136651 | | 14.66x | | 40.78 | | 123145 |
| Ln_09 | 103340 | | 14.78x | | 36.13 | | 94349 |
| Ln_10 | 130936 | | 15.03x | | 40.21 | | 118654 |
| Ln_11 | 87972 | | 14.39x | | 31.09 | | 80080 |
| Ln_12 | 20424 | | 16.81x | | 39.65 | | 19127 |
| Ln_13 | 134730 | | 14.15x | | 35.99 | | 121522 |
| Ln_14 | 77680 | | 15.46x | | 45.34 | | 71321 |
| Ln_15 | 107137 | | 15.40x | | 43.66 | | 97417 |
| Ln_16 | 63955 | | 14.97x | | 37.16 | | 59622 |
| Ln_17 | 61399 | | 15.88x | | 45.06 | | 56765 |
| Ln_18 | 74428 | | 15.65x | | 44.28 | | 68968 |
| Ln_20 | 123234 | | 14.85x | | 40.19 | | 110857 |
| G_01 | 299382 | | 12.59x | | 19.87 | | 266816 |
| G_02 | 387750 | | 13.15x | | 22.96 | | 341533 |
| G_03 | 538878 | | 13.49x | | 19.32 | | 467182 |
| G_04 | 362585 | | 13.23x | | 21.19 | | 316375 |
| G_05 | 902216 | | 16.70x | | 21.76 | | 784885 |
| G_06 | 437696 | | 13.54x | | 19.08 | | 379805 |
| G_07 | 387603 | | 13.66x | | 23.11 | | 336985 |
| G_08 | 936258 | | 17.64x | | 23.29 | | 802607 |
| G_09 | 669693 | | 14.33x | | 24.28 | | 582426 |
| G_10 | 477988 | | 13.87x | | 27.53 | | 417571 |
| G_11 | 451902 | | 13.52x | | 22.58 | | 392457 |
| G_12 | 387464 | | 13.39x | | 23.8 | | 338993 |
| G_13 | 551444 | | 13.55x | | 19.47 | | 474771 |
| G_14 | 686344 | | 14.52x | | 18.77 | | 592470 |
| G_15 | 437708 | | 13.26x | | 22.22 | | 386604 |
| G_16 | 540480 | | 14.11x | | 19.64 | | 467963 |
| G_17 | 416715 | | 13.45x | | 20.47 | | 361420 |
| G_18 | 193817 | | 13.09x | | 24.62 | | 172484 |
| G_20 | 400128 | | 13.65x | | 25.98 | | 352495 |
| P_01 | 266071 | | 14.15x | | 23.29 | | 235502 |
| P_02 | 260713 | | 15.14x | | 32.37 | | 230025 |
| P_03 | 245363 | | 14.47x | | 26.66 | | 216849 |
| P_04 | 247488 | | 14.30x | | 26.88 | | 216813 |
| P_05 | 322125 | | 14.56x | | 30.32 | | 281223 |
| P_06 | 353150 | | 14.49x | | 24.59 | | 305397 |
| P_07 | 730181 | | 18.24x | | 28.22 | | 622601 |
| P_08 | 206269 | | 14.54x | | 32.97 | | 182253 |
| P_09 | 230639 | | 14.54x | | 29.49 | | 202906 |
| P_10 | 445028 | | 15.53x | | 27.61 | | 383228 |
| P_11 | 290242 | | 14.43x | | 27.22 | | 252432 |
| P_12 | 196074 | | 14.06x | | 24.96 | | 172736 |
| P_13 | 165105 | | 14.18x | | 27.72 | | 146437 |
| P_14 | 1027322 | | 21.49x | | 28.04 | | 865193 |
| P_15 | 310109 | | 14.09x | | 24.75 | | 268835 |
| P_16 | 269357 | | 14.46x | | 24.14 | | 233199 |
| P_17 | 329946 | | 14.27x | | 25.95 | | 282240 |
| P_18 | 313834 | | 14.73x | | 24.99 | | 268470 |
| P_19 | 378404 | | 15.52x | | 28.24 | | 318887 |
| P_20 | 339290 | | 14.96x | | 25.61 | | 283201 |

**Table S3.** Pairwise F_ST_ values for L. nelumbifolia, C. stenoglossum and hybrids population (combines morphotypes G&P) based on 2,540 SNPs.

|  | C. stenoglossum | morphotype G&P | L. nelumbifolia |
| --- | --- | --- | --- |
| C. stenoglossum | * | 0.00000+-0.0000 | 0.00000+-0.0000 |
| morphotype G&P | 0.24103 | * | 0.00000+-0.0000 |
| L. nelumbifolia | 0.71751 | 0.25374 | * |
